# Small vs. Large Unruptured Cerebral Aneurysm: Concerns With the Age of Patient

**DOI:** 10.3389/fneur.2021.735456

**Published:** 2021-09-21

**Authors:** Jianfeng Zheng, Ru Xu, Xiaochuan Sun, Xiaodong Zhang

**Affiliations:** Department of Neurosurgery, the First Affiliated Hospital of Chongqing Medical University, Chongqing, China

**Keywords:** unruptured aneurysms, age, treatment, complications, outcomes

## Abstract

**Objective:** The coiling and clipping of unruptured cerebral aneurysms (UCAs) in older patients has increased rapidly, and aneurysm size was a significant factor for decision-making in the treatment of UCAs. The purpose of the study was to investigate the impact of age on the functional outcomes of patients between the small versus large UCAs.

**Methods:** We conducted a retrospective study for consecutive cases of UCAs admitted from May 2011 to December 2020. According to the maximum diameter of UCA, patients were divided into small UCAs (≤ 5 mm) group and large UCAs (>5 mm) group. Baseline characteristics, clinical complications, and outcomes of patients between the two groups were analyzed.

**Results:** A total of 564 UCA patients received preventive treatment, including 165 small UCAs and 399 large UCAs. Compared with the small UCA group, the incidence of ischemia event in the large UCA group was significantly higher (7.3 vs. 2.4%; *p* = 0.029). Multivariable analysis demonstrating age (*p* = 0.006) and treatment modality (*p* < 0.001) were independent risk factors associated with poor outcome for patients with large UCAs.

**Conclusions:** Preventive treatment of small UCAs is safe and effective, but older patients with large UCAs are at high risk of poor outcome, and the operations should be more cautious.

## Introduction

The prevalence of unruptured cerebral aneurysms (UCAs) varies from 2.3% to 7.0% in Chinese population (1, 2), and the older UCA patients have increasingly been diagnosed in the last few decades due to development of imaging technology and steadily aging society (3, 4). Because of vasculature tortuosity and sclerosis, more comorbidities, and shorter life expectancy (5, 6), the optimal management of older patients with UCAs remains controversial and challenging. Aneurysm size was a significant factor for decision-making in the treatment of UCAs. However, no studies have reported differences in outcomes in patients of different ages after prevention treatment of small UCAs vs. large UCAs (6, 7). The poor outcome occurred at a significantly greater rate due to higher risk of operation and anesthesia, and postoperative complications, leading to neurosurgeons and neurointerventionist unwilling to provide preventive intervention for older patients with asymptomatic small UCAs. On the other hand, the small UCAs carry a risk of rupture which can lead to intracranial hematoma (ICH), intraventricular hemorrhage (IVH), subarachnoid hemorrhage (SAH), and subsequent severe disability or even death in older patients (7–9). Therefore, the purpose of this study is to investigate the effect of age on the functional outcomes of patients between the small and large UCAs in order to better risk-stratify management and benefit more patients with UCAs.

## Patients and Methods

Between May 2011 and December 2020, we analyzed the clinical and imaging data of consecutive patients with UCAs hospitalized. Detailed information was collected, including age, sex, smoking history, drinking history, hypertension, diabetes, hyperlipidemia, etc. The reasons why the aneurysm was detected for all patients were investigated, such as oculomotor nerve palsy, visual loss, dizziness, headache, etc. Inclusion criteria: (1) The patient with a single or multiple UCAs who received clipping or coiling. (2) The patient was 18 years old and over. (3) The patient with complete clinical and imaging data. Exclusion criteria: (1) The patient who were treated with microsurgery or endovascular treatment in other hospitals during follow-up. (2) The patient with aneurysms associated with arteriovenous malformations, moyamoya disease, or other vascular diseases. (3) The patient with blister aneurysms, dissecting aneurysms, or giant aneurysms larger than 25 mm. On the basis of three-dimensional angiography, characteristics such as the location, size, and number of unruptured aneurysms were analyzed. According to the maximum diameter of aneurysms, patients were divided into small (≤ 5 mm) and large (>5 mm) UCA group. Endovascular or microsurgical treatment was performed *via* consensus of the neurosurgeons and neurointerventionists after carefully evaluating each UCA patient's condition. Considering the operation for unruptured aneurysm, all patients were informed in detail about the risk of treatment. Neurological complications including hemorrhagic event, ischemia event, and intracranial infection were assessed by two professional neurosurgeons. Bleeding events include intraoperative bleeding, postoperative hematoma, and postdischarge hemorrhage. Ischemic events include postoperative symptomatic ischemia and postdischarge symptomatic infarction. Systemic complications including pulmonary infection, urinary infection, and venous thrombosis were evaluated by the relevant department physicians. Imaging follow-up was performed by CTA at 3 months and DSA at 6 months after discharge. At the last telephone or outpatient follow-up, modified Rankin Scale (mRS) score was calculated for each patient, and those with mRS score ≥3 were considered poor outcomes.

### Statistical Analysis

Data related to categorical variables were presented as percentages, and Fisher's exact test or Pearson's χ^2^ test was used to compare female, smoking history, hypertension, diabetes, hyperlipidemia, cardiopulmonary disease, ischemic cerebrovascular disease, treatment reasons, aneurysm location, multiple aneurysms, treatment modality, complication, and other factors. Data related to continuous variables are presented as the mean ± SD if it conforms to normal distribution, and Student's *t*-test or Mann-Whitney *U*-test was used to compare age, size of largest aneurysm, and length of hospital stay. Univariate and multivariate logistic regression analyses were performed to determine the risk factors significantly associated with poor outcome. Variables with *p* < 0.1 by univariate analysis were entered into the multivariate analysis model. Statistical analysis was performed using SPSS V.22 software (IBM Corp., Armonk, New York, USA), and results with *P* < 0.05 were considered to indicate statistical significance.

## Result

During the study period, a total of 564 patients were admitted in our center, including 165 (29.3%) small UCAs and 399 (70.7%) large UCAs. The baseline characteristics, including sex, age, smoking history, drinking history, hypertension, diabetes, hyperlipidemia, previous history of cerebral infarction, and previous history of SAH were similar between the small and large UCA groups (Table 1). Among the reasons why the aneurysm was detected, oculomotor nerve palsy accounted for 12.2%, vision loss for 4.8%, gradual increase of aneurysm size for 1.1%, history of prior treatment for 4.1%, and headache, dizziness, or limb weakness for 58.2%. More patients with large UCAs underwent preventive treatment due to cranial nerve symptoms (15.8 vs. 3.6%; *p* < 0.001), while more patients with small UCAs underwent preventive treatment due to history of prior treatment (7.9 vs. 2.5%; *p* = 0.008) (Table 1).

**Table 1:** Clinical characteristics of patients with UCAs, divided into small and large UCA groups.

	**Total**	**Small UCA group**	**Large UCA group**	***P*-value**
	**(*n* = 564)**	**(*n* = 165)**	**(*n* = 399)**	
Sex (female)	389 (69.0%)	103 (62.4%)	286 (71.7%)	0.036
Age (years)	55.6 ± 10.6	54.3 ± 10.7	56.2 ± 10.5	0.052
Smoking history	98 (17.4%)	34 (20.6%)	64 (16.0%)	0.222
Drinking history	76 (13.5%)	28 (17.0%)	48 (12.0%)	0.136
Hypertension	249 (44.1%)	68 (41.2%)	181 (45.4%)	0.402
Diabetes	41 (7.3%)	13 (7.9%)	28 (7.0%)	0.723
Hyperlipidemia	43 (7.6%)	14 (8.5%)	29 (7.3%)	0.605
Previous history of CI	25 (4.4%)	9 (5.5%)	16 (4.0%)	0.501
Previous history of SAH	21 (3.7%)	9 (5.5%)	12 (3.0%)	0.219
**Detection reasons**				
Oculomotor nerve palsy	69 (12.2%)	6 (3.6%)	63 (15.8%)	<0.001
Visual loss	27 (4.8%)	4 (2.4%)	23 (5.8%)	0.127
Gradually increase in size	6 (1.1%)	2 (1.2%)	4 (1.0%)	1.000
History of prior treatment	23 (4.1%)	13 (7.9%)	10 (2.5%)	0.008
Other symptoms	328 (58.2%)	93 (56.4%)	235 (58.9%)	0.639

*CI, cerebral infarction; SAH, subarachnoid hemorrhage. Other symptoms include headache, dizziness, or limb weakness. Results with p < 0.05 were considered to indicate statistical significance*.

Seven hundred and eleven UCAs were diagnosed by 3D-CTA in 564 patients, including 194 (27.3%) aneurysms in small UCA group and 517 (72.7%) aneurysms in large UCA group. The proportion of AcomA and AcA aneurysms in the small UCA group was significantly higher than that in the large UCA group (*p* < 0.001; *p* = 0.007), and there was no significant difference in other locations of aneurysms (Table 2). In the small UCA group, the average diameter of the largest aneurysm was 3.8 mm, and 25 (12.9%) aneurysms were irregularly shaped; in the large UCA group, the average diameter of the largest aneurysm was 9.8 mm, and 77 (14.9%) aneurysms were irregularly shaped. Besides, 26 (13.4%) patients in the small UCA group had multiple aneurysms and 100 (19.3%) in the large UCA group had multiple aneurysms (Table 2).

**Table 2:** Location and characteristics of UCAs, divided into small and large UCA groups.

	**Total**	**Small UCA group**	**Large UCA group**	***P*-value**
	**(*n* = 564)**	**(*n* = 165)**	**(*n* = 399)**	
Number of aneurysms	711 (100%)	194 (27.3%)	517 (72.7%)	–
**Aneurysm location**				
AcomA	62 (8.7%)	30 (15.5%)	32 (6.2%)	<0.001
PcomA	151 (21.2%)	32 (16.5%)	119 (23.0%)	0.064
MCA	60 (8.4%)	16 (8.2%)	44 (8.5%)	1.000
ACA	8 (1.1%)	6 (3.1%)	2 (0.4%)	0.007
ICA	385 (54.1%)	102 (52.6%)	283 (54.7%)	0.613
PC	45 (6.3%)	8 (4.1%)	37 (7.2%)	0.167
**Aneurysm characteristics**				
Irregular shape	102 (14.3%)	25 (12.9%)	77 (14.9%)	0.549
Multiple aneurysms	126 (17.7%)	26 (13.4%)	100 (19.3%)	0.077
Diameter of largest aneurysm	8.1 ± 5.9	3.8 ± 1.0	9.8 ± 6.2	<0.001

*AcomA, anterior communicating artery; PcomA, posterior communicating artery; MCA, middle cerebral artery; ACA, anterior cerebral artery; ICA, internal carotid artery; PC, posterior circulation artery. Results with p < 0.05 were considered to indicate statistical significance*.

### Clinical Complications

**Figure 1:**
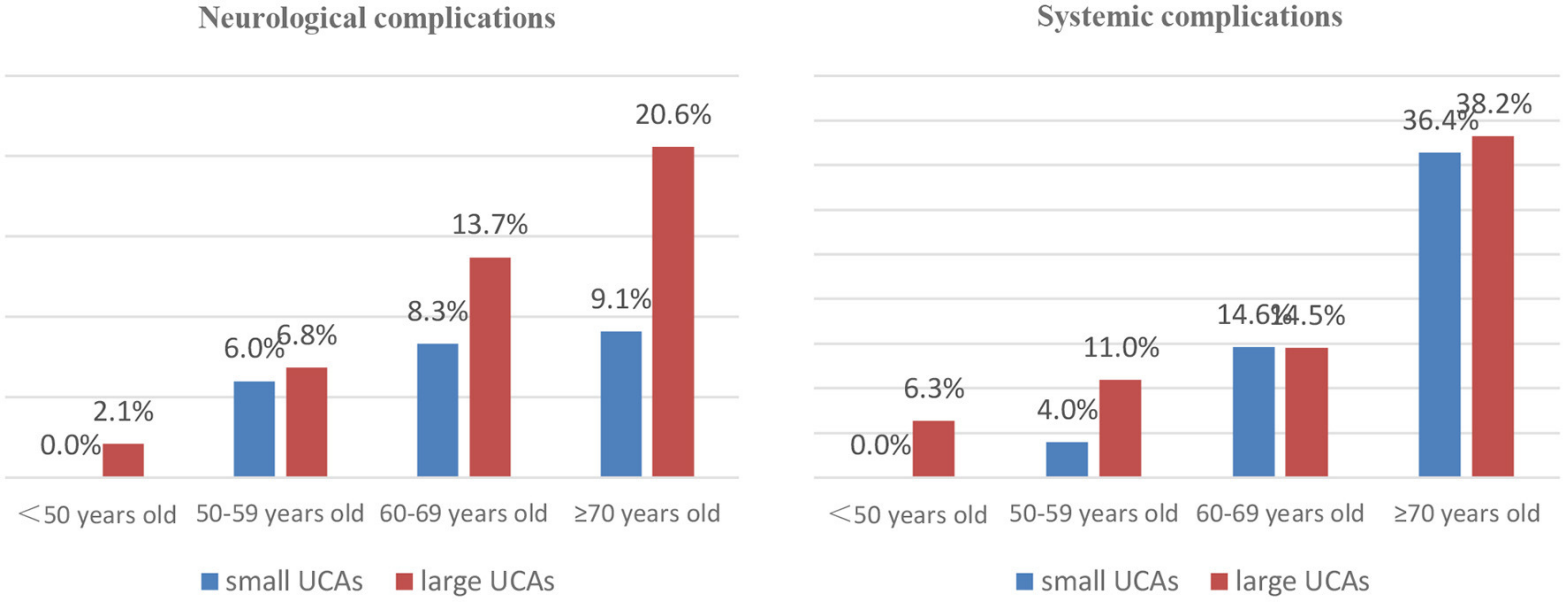
The distribution of neurological and systemic complications according to age of patients.

In the small UCA group, 45 patients were treated with clipping, 25 were embolized with detachable coils, and 95 were treated with stent-assisted coiling. In the large UCA group, 86 patients were treated with clipping, 39 were embolized with detachable coils, 264 were treated with stent-assisted coiling, and 10 were treated with balloon-assisted coiling. There was no significant difference in treatment modalities between the two groups (Table 3). Compared with the small UCA group, the incidence of ischemia event in the large UCA group was significantly higher (7.3 vs. 2.4%; *p* = 0.029). However, the incidences of hemorrhagic event, intracranial infection, pulmonary infection, pulmonary embolism, urinary infection, and venous thrombosis were similar between the two groups (Table 3). With the increase of age, the incidence of neurological and systemic complications increases in patients with large UCAs, but there was no such trend in the small UCA group (Figure 1).

**Table 3:** Complications and outcome of patients with UCAs, divided into small and large UCA groups.

	**Total**	**Small UCA group**	**Large UCA group**	***P*-value**
	**(*n* = 564)**	**(*n* = 165)**	**(*n* = 399)**	
Microsurgical clipping	131 (23.2%)	45 (27.3%)	86 (21.6%)	0.155
Endovascular coiling	433 (76.8%)	120 (72.7%)	313 (78.4%)	
Neurological complications				
Hemorrhagic event	10 (1.8%)	3 (1.8%)	7 (1.8%)	1.000
Ischemia event	33 (5.9%)	4 (2.4%)	29 (7.3%)	0.029
Intracranial infection	19 (3.4%)	4 (2.4%)	15 (3.8%)	0.609
Systemic complications				
Pulmonary infection	56 (9.9%)	10 (6.1%)	46 (11.5%)	0.062
Pulmonary embolism	2 (0.4%)	0 (0.0%)	2 (0.5%)	1.000
Urinary infection	7 (1.2%)	1 (0.6%)	6 (1.5%)	0.680
Venous thrombosis	18 (3.2%)	3 (1.8%)	15 (3.8%)	0.299
Good outcome	542 (96.1%)	162 (98.2%)	380 (95.2%)	0.149
mRS 0	511 (90.6%%)	153 (92.7%)	358 (89.7%)	0.341
mRS 1	26 (4.6%)	7 (4.2%)	19 (4.8%)	1.000
mRS 2	5 (0.9%)	2 (1.2%)	3 (0.8%)	0.633
Poor outcome	22 (3.9%)	3 (1.8%)	19 (4.8%)	0.149
mRS 3	5 (0.9%)	2 (1.2%)	3 (0.8%)	0.633
mRS 4	6 (1.1%)	1 (0.6%)	5 (1.3%)	0.677
mRS 5	6 (1.1%)	0 (0%)	6 (1.5%)	0.188
mRS 6	5 (0.9%)	0 (0%)	5 (1.3%)	0.328

*mRS, modified Rankin Scale. Results with p < 0.05 were considered to indicate statistical significance*.

### Outcomes and Risk Factors

**Figure 2:**
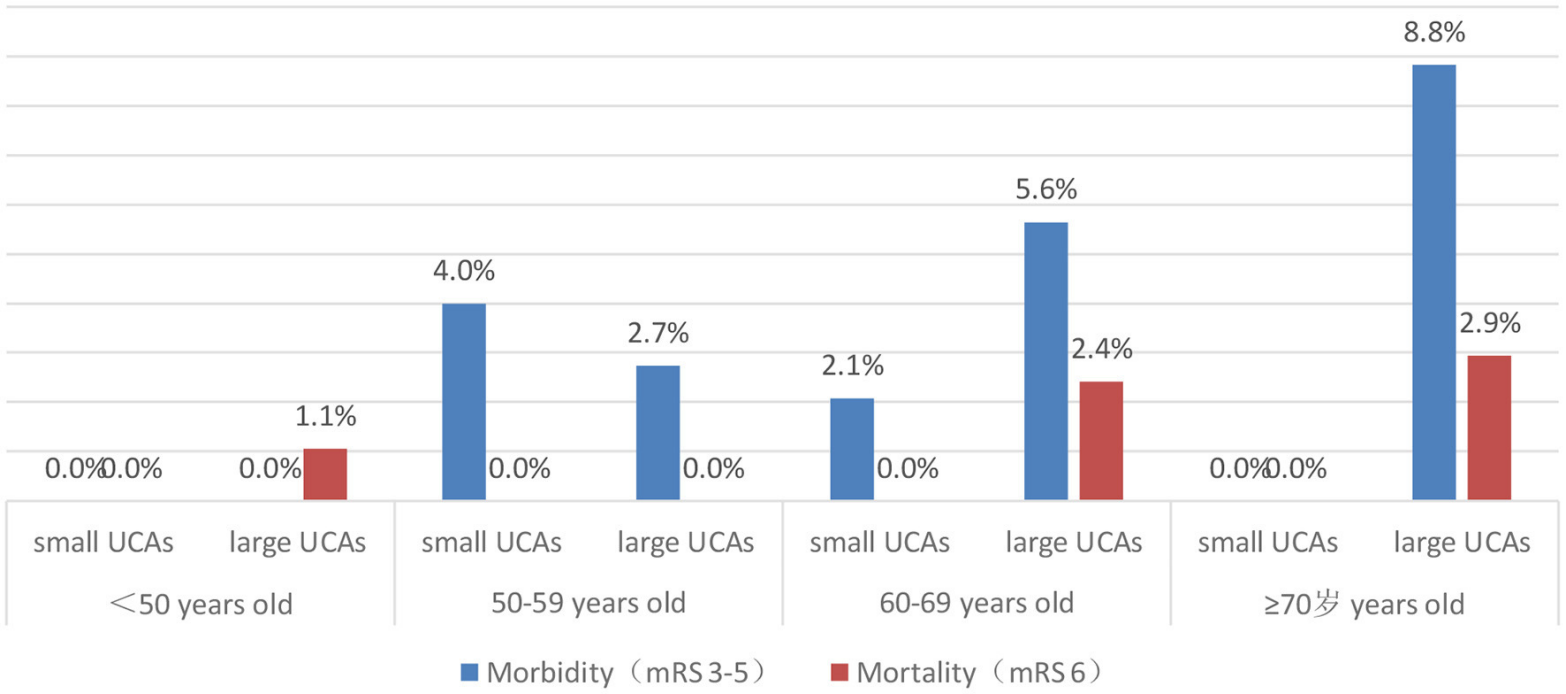
The distribution of morbidity and mortality according to age of patients.

After 3–66 months of follow-up (mean 17 months), 96.1% of patients had a good outcome (mRS 0-2). The good outcome rate of the small UCA group was higher than that of the large UCA group, but it is not statistically significant (98.2 vs. 95.2%; *p* = 0.149). A univariate analysis was performed to identify risk factors associated with poor outcome for patients with large UCAs. In univariate analysis, age (*p* = 0.001) and hypertension (*p* = 0.046), hyperlipidemia (*p* = 0.056) and treatment modality (*p* = 0.002) were obviously associated with poor outcomes for patients with large UCAs. After adjustment for these covariates, multivariable analysis demonstrating age [*p* = 0.006; OR, 1.090 (1.025–1.158)] and treatment modality [*p* < 0.001; OR, 6.177 (2.209–17.273)] were independent risk factors associated with poor outcome for patients with large UCAs (Table 4). In the large UCA group, the morbidity and mortality increased with the increase of age, but there was no such trend in the small UCA group (Figure 2). Angiography reexamination (CTA, MRA, or DSA) of 3 months or more was achieved in 348 (61.7%) patients. There was no recurrence of aneurysm in the small UCA group, while five cases (1.3%) had recurrence of aneurysm in the large UCA group and three cases (0.8%) were retreated with endovascular treatment.

**Table 4:** Risk factors associated with poor outcome in patients with UCAs.

	**Small UCA group**	**Large UCA group**		
	**Univariate analysis**	**Univariate analysis**	**Multivariable analysis**	
	***P*-value**	***P*-value**	***P*-value**	**OR (95% CI)**
Female sex	0.997	0.474	–	
Age	0.697	0.001	0.006	1.090 (1.025–1.158)
Smoking history	0.998	0.219	–	
Drinking history	0.998	0.837	–	
Hypertension	0.387	0.046	0.149	
Hyperlipidemia	0.999	0.056	0.076	
Diabetes	0.999	0.543	–	
Previous history of CI	0.999	0.405	–	
Previous history of SAH	0.999	0.999	–	
Size of largest aneurysm	0.311	0.330	–	
Location of largest aneurysm	0.524	0.488	–	
Multiple aneurysms	0.996	0.504	–	
Treatment (coiling vs. clipping)	0.996	0.002	<0.001	6.177 (2.209–17.273)

*Variables with p < 0.1 according to univariate analysis were entered into the multivariate analysis model. OR, odds ratio; CI, confidence interval. Results with p < 0.05 were considered to indicate statistical significance*.

## Discussion

The accelerated aging of the world population has a great impact on the incidence and prevalence of cerebrovascular disease, so more patients with UCAs were detected and treated in the last few decades (1–4). One hundred sixty-five patients with small UCAs were admitted to our center in 10 years, accounting for 29.3% of all treated UCAs in the same period. Clinical management of UCAs is greatly challenging because of the low risk of rupture and high risk of operation. However, the effect of age on the functional outcome of patients after prevention treatment of the small UCAs vs. large UCAs is still unclear. Therefore, we analyzed all UCA patients and compared the complications of small UCAs and large UCAs and the outcomes of patients of different ages. In the small UCA group, the preventive treatment is safe and effective for both young and older patients; however, in the large UCA group, all older patients had more treatment-related complications and are at high risk of poor outcomes.

The treatment mode of UCAs has undergone tremendous changes in the last 20 years (9–14); most older aneurysm patients in history have been excluded from surgery due to high risk of operation. Since the advent of revolutionary intravascular technology, more UCAs have been aggressively treated with endovascular coiling, especially in the older patients with decreased function of body organs. Barker et al. (9) first conducted a retrospective analysis using the sample data of inpatients in the USA from 1996 to 2000 and reported that the mortality rates were similar after clipping or coiling of UCAs. However, from the sample data between 2000 and 2006 (10), the coiling of UCAs was obviously associated with fewer deaths and perioperative complications compared with clipping. Brinjikji et al. (11) similarly reported that the morbidity and mortality of patients treated with coiling were significantly lower than that of patients treated with clipping, and these differences become more obvious with age. Therefore, if treatment is deemed necessary for older patients with UCAs, endovascular treatment may be the best option (12). According to a recent study on older patients with UCAs, the mortality rate of clipping was similar to that of coiling (13). The main reason for this change of mortality is that microsurgical clipping has become safer and more tolerable in older UCA patients provided with advanced intraoperative neuromonitoring and neurointensive care. Consistent with these studies, in our study, the patients treated by endovascular treatment accounted for approximately 76.8% of all UCA patients. There is no significant difference in mortality between coiling and clipping in both the small and large UCA patients. In addition to age, aneurysm size was also significantly associated with outcomes in patients with UCAs. Feng et al. (15) reported that the morbidity and mortality of 264 patients with small UCAs treated by coiling were 2.7 and 0.9%, while the morbidity and mortality of our patients with small UCAs were 1.8 and 0%. The morbidity and mortality of our patients with large UCAs were 3.6 and 1.3%. In particular, patients over 70 years of age with large UCAs are at higher risk of complications and poor outcomes.

Increasing age has an important impact on the treatment outcomes of patients with UCAs (5, 16). Meanwhile, age itself is reported to be a significant risk factor for the growth and rupture of aneurysm (4, 17). Wermer et al. (18) believed that the rupture risk of UCAs is obviously increased in the patients over 60 years old. Nevertheless, there are different opinions on which patients are suitable for preventive surgery rather than for observation conservatively. Some UCA patients died after surgical or endovascular treatment due to more procedural complications, and some died of aneurysm rupture during conservative follow-up. Our study found that the neurological complications and poor outcomes are more common in patients over 60 years old in large UCA group, but the morbidity and mortality did not increase with age in the small UCA group. The assumption that unruptured aneurysms should be conservatively observed is based on the low risk of rupture or low morbidity and mortality (19, 20). Aishima et al. (21) and Malhotra et al. (22) believed that patients with UCAs or small UCAs without risk factors of rupture should be observed conservatively. In fact, the ISUIA and the UCAS studies shows that the risk of aneurysm rupture in older patients is obviously increased (16, 20). Also, older patients are more likely to suffer from ischemic-related complications after aneurysm rupture due to age-related physiological changes, such as hardening of vessels and increased vessel tortuosity leading to diminished ability to repair microinjuries acquired from wall shear stresses (23, 24). Our study showed that the morbidity and mortality of patients with small UCAs are very low after surgical or endovascular treatment, so preventive interventions for older patients with small UCAs is safe and feasible, and it also avoid the high risk of fatal bleeding for older patients with small UCAs.

The unruptured intracranial aneurysm treatment score (UIATS), developed and released by multidisciplinary experts, was mainly used to guide the clinical management and decision-making of patients with UCAs (25). Several studies (26–28) then reported that the UIATS scale is relatively complex and lacks effective verification, and its clinical application is still controversial. A study conducted by Smedley et al. (26) validated 296 cases of UCAS and reported that their expert opinion is consistent with that of the UIATS, but differences remain between the two. In a retrospective analysis of 93 patients with 147 UCAs, Hernández-Durán et al. (27) thought that the decision to treat UCAs remains a personalized one, without a reliable predictor, and one should employ the UIATS with caution. Meanwhile, in a single-center study comparing UIATS with actual clinical experience, Ravindra et al. (28) found that the UIATS usually recommended overtreatment of unruptured aneurysms. Similarly, the UIATS scale has not been applied for the treatment decision of UCAs in our center, especially considering the underdeveloped economic and medical level of Western China. UCA management guidelines (29) show that aneurysm that causes any neurological symptoms should be actively treated regardless of the size. In this study, most of the patients (76.3%) who received preventive treatment had obvious symptoms, while a few of the small UCA patients without obvious symptoms also had different levels of anxiety. Some studies also believe that psychological stress is a potential risk factor for hemorrhagic stroke, and the sudden rise of blood pressure due to fear is significantly related to aneurysm rupture (30–32). Small UCA patients have better treatment outcomes and lower morbidity and mortality; however, the poor outcome rate of older patients with large UCAs is high, and the treatment indications of these patients should be more cautious. The overtreatment of UCAs should be avoided when the benefits of preventive treatment are not fully identified as greater than that of conservative observation. Few previous studies have focused on comparing the treatment outcome of UCAs based on patient age and aneurysm size, and most of these previous studies have focused on the treatment outcome of coiling or clipping for UCAs (14, 33, 34). This is the first study to compare the effect of age on outcomes of patients between the small UCAs and large UCAs, so this is the strengths and uniqueness. Nevertheless, there are several limitations in the current study. First, the number of older patients with UCAs who received prevention treatment is small due to the low life expectancy and the economic development in Western China. Second, this study is a single-center retrospective design, so a large prospective study with complete follow-up should be conducted in the future.

## Conclusion

Endovascular coiling and microsurgical clipping are effective remedy techniques for UCAs, and small UCA patients with high risk of rupture can benefit from preventive intervention. However, we believed that the indications of treatment for large UCAs should be carefully evaluated especially in older patients who are at high risk of poor outcome after preventive treatment of large UCAs.

## Data Availability Statement

The original contributions presented in the study are included in the article/supplementary material, further inquiries can be directed to the corresponding author/s.

## Ethics Statement

The studies involving human participants were reviewed and approved by Ethics Committee of the First Affiliated Hospital of Chongqing Medical University. Written informed consent for participation was not required for this study in accordance with the national legislation and the institutional requirements.

## Author Contributions

All authors listed have made a substantial, direct and intellectual contribution to the work, and approved it for publication.

## Funding

This study was supported by the National Natural Science Foundation of China (No. 81571159) and National Key Research Development Program (grants no 2016YFC1300800).

## Conflict of Interest

The authors declare that the research was conducted in the absence of any commercial or financial relationships that could be construed as a potential conflict of interest.

## Publisher's Note

All claims expressed in this article are solely those of the authors and do not necessarily represent those of their affiliated organizations, or those of the publisher, the editors and the reviewers. Any product that may be evaluated in this article, or claim that may be made by its manufacturer, is not guaranteed or endorsed by the publisher.

## References

[1] Chan DY Abrigo JM Cheung TC Siu DY Poon WS Ahuja AT. Screening for intracranial aneurysms? Prevalence of unruptured intracranial aneurysms in Hong Kong Chinese. J Neurosurg. (2016) 124:1245–9. 10.3171/2015.4.JNS142938 26473778

[2] Li MH Chen SW Li YD Chen YC Cheng YS Hu DJ. Prevalence of unruptured cerebral aneurysms in Chinese adults aged 35 to 75 years: a cross-sectional study. Ann Intern Med. (2013) 159:514–21. 10.7326/0003-4819-159-8-201310150-00004 24126645

[3] Vlak MH Algra A Brandenburg R Rinkel GJ. Prevalence of unruptured intracranial aneurysms, with emphasis on sex, age, comorbidity, country, and time period: a systematic review and meta-analysis. Lancet Neurol. (2011) 10:626–36. 10.1016/S1474-4422(11)70109-0 21641282

[4] Harada K Fukuyama K Shirouzu T Ichinose M Fujimura H Kakumoto K. Prevalence of unruptured intracranial aneurysms in healthy asymptomatic Japanese adults: differences in gender and age. Acta Neurochir. (2013) 155:2037–43. 10.1007/s00701-013-1841-7 23959131

[5] Khosla A Brinjikji W Cloft H Lanzino G Kallmes DF. Age-related complications following endovascular treatment of unruptured intracranial aneurysms. AJNR Am J Neuroradiol. (2012) 33:953–7. 10.3174/ajnr.A2881 22241386 PMC7968821

[6] Higashida RT Lahue BJ Torbey MT Hopkins LN Leip E Hanley DF. Treatment of unruptured intracranial aneurysms: a nationwide assessment of effectiveness. AJNR Am J Neuroradiol. (2007) 28:146–51. 17213445 PMC8134123

[7] Kwinta BM Kliś KM Krzyzewski RM Wilk A Dragan M Grzywna E. Elective management of unruptured intracranial aneurysms in elderly patients in a high-volume center. World Neurosurg. (2019) 126: e1343–51. 10.1016/j.wneu.2019.03.094 30898743

[8] de Rooij NK Linn FH van der Plas JA Algra A Rinkel GJ. Incidence of subarachnoid haemorrhage: a systematic review with emphasis on region, age, gender and time trends. J Neurol Neurosurg Psychiatry. (2007) 78:1365–72. 10.1136/jnnp.2007.117655 17470467 PMC2095631

[9] Barker FG 2nd Amin-Hanjani S Butler WE Hoh BL Rabinov JD Pryor JC. Age-dependent differences in short-term outcome after surgical or endovascular treatment of unruptured intracranial aneurysms in the United States, 1996–2000. Neurosurgery. (2004) 54:18–28. 10.1227/01.NEU.0000097195.48840.C4 14683537

[10] Alshekhlee A Mehta S Edgell RC Vora N Feen E Mohammadi A. Hospital mortality and complications of electively clipped or coiled unruptured intracranial aneurysm. Stroke. (2010) 41:1471–6. 10.1161/STROKEAHA.110.580647 20522817

[11] Brinjikji W Rabinstein AA Lanzino G Kallmes DF Cloft HJ. Effect of age on outcomes of treatment of unruptured cerebral aneurysms: a study of the National Inpatient Sample 2001–2008. Stroke. (2011) 42:1320–4. 10.1161/STROKEAHA.110.607986 21441142

[12] Mahaney KB Brown RD Jr Meissner I Piepgras DG Huston J 3rd Zhang J. Age-related differences in unruptured intracranial aneurysms: 1-year outcomes. J Neurosurg. (2014) 121:1024–38. 10.3171/2014.6.JNS121179 25170670

[13] Ikawa F Michihata N Akiyama Y Iihara K Matano F Morita A. Treatment risk for elderly patients with unruptured cerebral aneurysm from a nationwide database in Japan. World Neurosurg. (2019) 132:e89–e98. 10.1016/j.wneu.2019.08.252 31518740

[14] Bekelis K Gottlieb DJ Su Y O'Malley AJ Labropoulos N Goodney P. Comparison of clipping and coiling in elderly patients with unruptured cerebral aneurysms. J Neurosurg. (2017) 126:811–8. 10.3171/2016.1.JNS152028 27203150 PMC5116411

[15] Feng X Wang L Guo E Zhang B Qian Z Liu P. Progressive occlusion and recanalization after endovascular treatment for 287 unruptured small aneurysms (<5 mm): a single-center 6-year experience. World Neurosurg. (2017). 126:811–8. 10.1016/j.wneu.2017.04.017 28416410

[16] Wiebers DO Whisnant JP Huston J 3rd Meissner I Brown RD Jr Piepgras DG. Unruptured intracranial aneurysms: natural history, clinical outcome, and risks of surgical and endovascular treatment. Lancet. (2003) 362:103–10. 10.1016/S0140-6736(03)13860-3 12867109

[17] Hishikawa T Date I Tokunaga K Tominari S Nozaki K Shiokawa Y. Risk of rupture of unruptured cerebral aneurysms in elderly patients. Neurology. (2015) 85:1879–85. 10.1212/WNL.0000000000002149 26511450 PMC4662696

[18] Wermer MJ van der Schaaf IC Algra A Rinkel GJ. Risk of rupture of unruptured intracranial aneurysms in relation to patient and aneurysm characteristics: an updated meta-analysis. Stroke. (2007) 38:1404–10. 10.1161/01.STR.0000260955.51401.cd 17332442

[19] Waqas M Rajabzadeh-Oghaz H Tutino VM Vakharia K Poppenberg KE Mowla A. Morphologic parameters and location associated with rupture status of intracranial aneurysms in elderly patients. World Neurosurg. (2019) 129:e831–7. 10.1016/j.wneu.2019.06.045 31207378

[20] UCAS Japan Investigators Morita A Kirino T Hashi K Aoki N Fukuhara S. The natural course of unruptured cerebral aneurysms in a Japanese cohort. N Engl J Med. (2012) 366:2474–82. 10.1056/NEJMoa1113260 22738097

[21] Aishima K Shimizu T Aihara M Yoshimoto Y. Lifetime effects of small unruptured intracranial aneurysms. World Neurosurg. (2016) 95:434–40. 10.1016/j.wneu.2016.08.060 27567575

[22] Malhotra A Wu X Forman HP Matouk CC Gandhi D Sanelli P. Management of tiny unruptured intracranial aneurysms: a comparative effectiveness analysis. JAMA Neurol. (2018) 75:27–34. 10.1001/jamaneurol.2017.3232 29159405 PMC5833486

[23] O'Neill AH Chandra RV Slater LA Chong W Xenos C Danks AR. Influence of comorbidities on treatment of unruptured intracranial aneurysms in the elderly. J Clin Neurosci. (2019) 62:38–45. 10.1016/j.jocn.2019.01.013 30655235

[24] Tawk RG Grewal SS Heckman MG Navarro R Ferguson JL Starke EL. Influence of body mass index and age on functional outcomes in patients with subarachnoid hemorrhage. Neurosurgery. (2015) 76:136–41. 10.1227/NEU.0000000000000588 25549185

[25] Etminan N Brown RD Jr Beseoglu K Juvela S Raymond J Morita A Torner JC. The unruptured intracranial aneurysm treatment score: a multidisciplinary consensus. Neurology. (2015) 85:881–9. 10.1212/WNL.0000000000001891 26276380 PMC4560059

[26] Smedley A Yusupov N Almousa A Solbach T Toma AK Grieve JP. Management of incidental aneurysms: comparison of single Centre multi-disciplinary team decision making with the unruptured incidental aneurysm treatment score. Br J Neurosurg. 2018; 32:536–40. 10.1080/02688697.2018.1468019 29764206

[27] Hernández-Durán S Mielke D Rohde V Malinova V. The application of the unruptured intracranial aneurysm treatment score: a retrospective, single-center study. Neurosurg Rev. (2018) 41:1021–8. 10.1007/s10143-018-0944-2 29388120

[28] Ravindra VM de Havenon A Gooldy TC Scoville J Guan J Couldwell WT. validation of the unruptured intracranial aneurysm treatment score: comparison with real-world cerebrovascular practice. J Neurosurg. (2018) 129:100–6. 10.3171/2017.4.JNS17548 28984518

[29] Thompson BG Brown RD Amin-hanjani S Broderick JP Cockroft KM Connolly ES Jr. Guidelines for the management of patients with unruptured intracranial aneurysms: a guideline for healthcare professionals from the American heart association/American stroke association. Stroke. (2015) 46:2368–400. 10.1161/STR.0000000000000070 26089327

[30] Henderson KM Clark CJ Lewis TT Aggarwal NT Beck T Guo H. Psychosocial distress and stroke risk in older adults. Stroke. (2013) 44:367–72. 10.1161/STROKEAHA.112.679159 23238864 PMC3552144

[31] Vlak MH Rinkel GJ Greebe P van der Bom JG Algra A. Trigger factors and their attributable risk for rupture of intracranial aneurysms: a case-crossover study. Stroke. (2011) 42:1878–82. 10.1161/STROKEAHA.110.606558 21546472

[32] Lee EJ Lee HJ Hyun MK Choi JE Kim JH Lee NR. Rupture rate for patients with untreated unruptured intracranial aneurysms in South Korea during 2006–2009. J Neurosurg. (2012) 117:53–9. 10.3171/2012.3.JNS111221 22519434

[33] Silva NA Shao B Sylvester MJ Eloy JA Gandhi CD. Unruptured aneurysms in the elderly: perioperative outcomes and cost analysis of endovascular coiling and surgical clipping. Neurosurg Focus. (2018) 44:E4. 10.3171/2018.1.FOCUS17714 29712518

[34] Qureshi AI Chaudhry SA Tekle WG Suri MF. Comparison of long-term outcomes associated with endovascular treatment vs surgical treatment among Medicare beneficiaries with unruptured intracranial aneurysms. Neurosurgery. (2014) 75:380–6. 10.1227/NEU.0000000000000450 24887287

